# ﻿Monograph of *Doselia* (Solanaceae), a new hemiepiphytic genus endemic to the northern Andes

**DOI:** 10.3897/phytokeys.202.82101

**Published:** 2022-07-26

**Authors:** Andrés Orejuela, Boris Villanueva, Clara Inés Orozco, Sandra Knapp, Tiina Särkinen

**Affiliations:** 1 Royal Botanic Garden Edinburgh, 20A Inverleith Row, Edinburgh, EH3 5LR, UK; 2 Herbario JBB, Subdirección Científica, Jardín Botánico de Bogotá José Celestino Mutis, Bogotá D.C., Colombia; 3 Max Planck Tandem Group, Facultad de Ciencias, Universidad Nacional de Colombia, Bogotá D.C., Colombia; 4 Instituto de Ciencias Naturales, Universidad Nacional de Colombia, Bogotá D.C., Colombia; 5 Department of Life Sciences, Natural History Museum, Cromwell Road, London SW7 5BD, UK

**Keywords:** Colombia, *Doselia*, Ecuador, endemism, hemiepiphytes, Juanulloeae, lianas, *Markea*, new species, Solandreae

## Abstract

A new genus, *Doselia* A.Orejuela & Särkinen, **gen. nov.**, is described in the tribe Solandreae (Solanaceae) consisting of four species of hemiepiphytic lianas endemic to the premontane forests of the Colombian and Ecuadorian Andes. The genus is distinguished based on the membranous leaves, usually sparsely pubescent with eglandular simple trichomes, pseudo-verticillate leaf arrangement, and elongated, pendulous, and few-flowered inflorescences with showy flowers and conical fruits. Three new combinations are made to transfer species to the new genus previously described as part of the polyphyletic genus *Markea* Rich. (*Doseliaepifita* (S.Knapp) A.Orejuela & Särkinen, **comb. nov.**, *D.huilensis* (A.Orejuela & J.M.Vélez) A.Orejuela & Särkinen, **comb. nov.** and *D.lopezii* (Hunz.) A.Orejuela & Särkinen, **comb. nov.**). One new species is described from the western slopes of the eastern cordillera of the Colombian Andes, known only from three localities in the Boyacá, Santander, and Tolima departments (*Doseliagalilensis* A.Orejuela & Villanueva, **sp. nov.**). The new species is unique in the genus in having glabrescent adult leaves, green-purplish calyces and long, greenish-white, infundibuliform corollas with delicate purplish veins and large lobes tinged with purple, and pubescent styles. Here we provide a revision of *Doselia* with a distribution map of all species, an identification key, photographs, preliminary conservation assessments, and line drawings of all four species.

## Abstract (es)

Un nuevo género, *Doselia* A.Orejuela & Särkinen, **gen. nov.**, es descrito en la tribu Solandreae (Solanaceae), conformado por cuatro especies de lianas hemiepífitas endémicas de los bosques premontanos de los Andes de Colombia y Ecuador. El género se distingue por sus hojas membranáceas con tricomas simples no glandulares, el arreglo pseudoverticilado de las hojas y sus inflorescencias paucifloras, largo pedunculadas y péndulas, flores vistosas y frutos cónicos. Se proponen tres nuevas combinaciones para acomodar especies previamente descritas en el polifilético género *Markea* Rich. (*Doseliaepifita* (S.Knapp) A.Orejuela & Särkinen, **comb. nov.**, *D.huilensis* (A.Orejuela & J.M.Vélez) A.Orejuela & Särkinen, **comb. nov.** and *D.lopezii* (Hunz.) A.Orejuela & Särkinen, **comb. nov.**) Una nueva especie es descrita aquí proveniente de la vertiente occidental de la cordillera Oriental de los Andes colombianos, y conocida únicamente de tres localidades en los departamentos de Boyacá, Santander y Tolima (*Doseliagalilensis* A.Orejuela & Villanueva, **sp. nov.**). La nueva especie se caracteriza por sus hojas glabrescentes cuando adultas, cáliz verde-púrpura, corolas infundibuliformes grandes, blanco-verdosas con una reticulación fina de venas púrpuras, lóbulos corolinos grandes y teñidos de púrpura en su lado ventral y estilos pubescentes. El nuevo género es formalmente descrito incluyendo un mapa de distribución, una clave taxonómica y fotografías e ilustraciones para sus cuatro especies.

## ﻿Introduction

The tribe Solandreae Miers (Solanaceae) contains ca. 80 species of mainly epiphytic or hemi-epiphytic lianas and shrubs in a number of genera currently being recircumscribed (Orejuela et al. 2017; Orejuela et al. in prep). The group is restricted to the Neotropics, with species distributed from Mexico and the Caribbean to Bolivia and southern Brazil (Orejuela et al. 2017). A centre of endemism for the tribe lies in Andean Ecuador and Colombia, where ca. 60% of the species are found (Orejuela et al. 2017).

The tribe Solandreae is a unique clade within Solanaceae in that many of its component taxa are epiphytic and hemiepiphytic plants with a great diversity of floral forms, pollinators, and ant associations. Epiphytes are rare in Solanaceae, with only ca. 90 species with this growth form across the family in three distinct tribes (Solandreae 80 spp.; Capsiceae 4–5 spp.; and Solaneae 4–5 spp.), with Solandreae containing most of the epiphytic species (ca. 90%; Hunziker 2001). The tribe is also the only group of Solanaceae with known ant associations (e.g., *Merinthopodium* Donn.Sm., *Markea* Rich., and species of *Hawkesiophyton* Hunz.; Knapp et al. 1997; Hunziker 2001; Orejuela et al. 2017).

Within Solandreae, there is notable morphological variation in corolla shape, size, and colour. Corollas vary from large infundibuliform or campanulate, long tubular, hypocrateriform to minutely campanulate and include pale or dull-coloured to brightly coloured forms. This remarkable variation suggests a diverse coevolutionary history with pollinators; bats, hummingbirds, and bees have all been observed to visit these flowers (Vogel 1958; Cocucci 1999; Muchhala and Jarrin-V 2002; Sazima et al. 2003; Knapp 2010).

Variation in floral form has been used as the basis of previous taxonomic classifications of the tribe. Molecular phylogenetic studies have shown, however, that many of the previously recognised genera in Solandreae are para- or polyphyletic and in dire need of taxonomic revision (Orejuela et al. 2017). In addition to extensive re-circumscription of genera, two new lineages have been identified within Solandreae based on nuclear and plastid Sanger sequences and whole plastome data that represent distinct morphological groups comprised of species previously described as members of *Markea* that are distinct at the generic level: the *Markealopezii* and *Markeaantioquiensis* clades (Orejuela et al. 2017; Orejuela et al. in prep).

**Figure 1.**
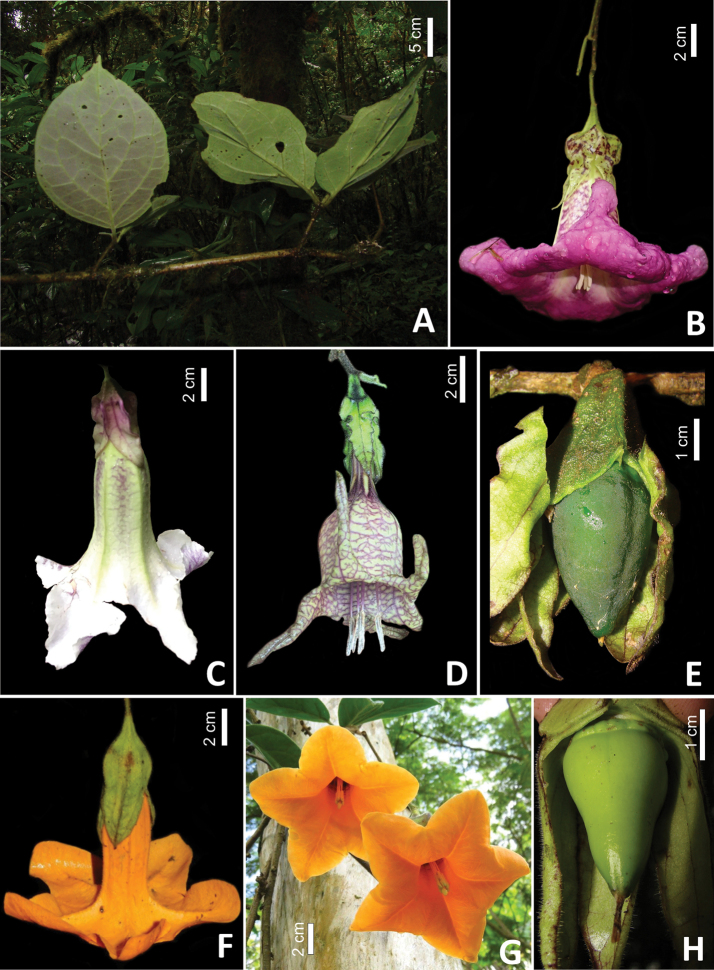
*Doselia* A.Orejuela & Särkinen **A** pseudoverticillate leaves in *D.epifita***B** infundibuliform cream-purplish corolla of *D.epifita***C** infundibuliform greenish-white corolla with subtle purple veins of *D.galilensis***D** tubular-campanulate yellowish-green corolla of *D.huilensis* with strong purple-tinged reticulation along major and minor veins **E** developing fruit in *D.huilensis***F** hypocrateriform orange corollas in *D.lopezii***G** pendent long flowering branches in *D.lopezii* with clustered leaves and orange corollas in frontal view **H** developing fruit in *D.lopezii* with a nectariferous disc at the base (Vouchers: **A***Orozco et al. 3876* (COL), **B** no voucher, **C***Corrales et al. 917* (JBB, TOLI), **D***Coral 34* (HEEA), **E***Orejuela & Vélez-Puerta* 112 (COL) **F***Orejuela et al. 727* (JBB) **G** no voucher **H***Orejuela & Calderon 170* (COL): photos by Alistair Hay, Andreas Kay, Andrés Orejuela, Boris Villanueva, Brayan Coral, and Eduardo Calderon.

**Figure 2.**
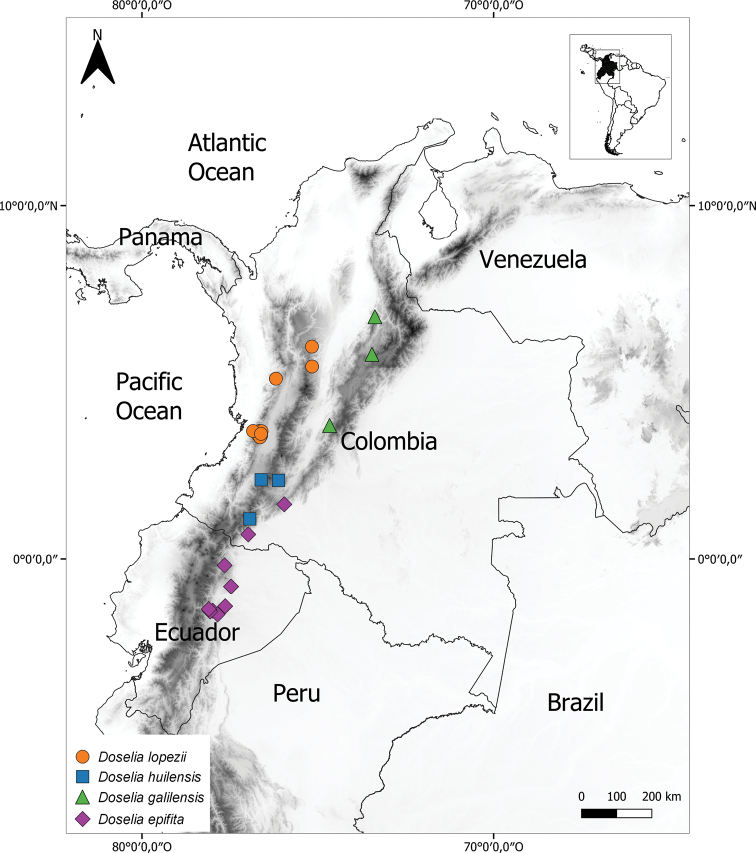
Geographic distribution of *Doselia*, including *D.galilensis* (green triangles).

Here we focus on the morphologically distinct *Markealopezii* clade (Figs 1, 2; Table 1), a group of four species from mid-elevation moist Andean forests of Ecuador and Colombia. The group includes three previously described species, *M.epifita* S.Knapp, *M.huilensis* A.Orejuela & J.M.Vélez, and *M.lopezii* Hunz. The fourth was discovered in 2018 during fieldwork in Colombia in the Parque Natural Regional Bosque de Galilea in the municipality of Villarrica, Tolima, and is described here. The four species treated here were resolved as a monophyletic group, named the *Markealopezii* clade, with strong branch support in a molecular phylogenomic study of Solandreae that included 95% of the species (76 spp.; Orejuela et al. in prep).

**Table 1.** Comparison of the new genus *Doselia* with the morphologically most closely related genera and groups in the tribe Solandreae (Solanaceae).

	*Doselia*	*Solandra*	*Schultesianthus*	*Markeaantioquiensis* clade
**Habit**	Hemiepiphytic lianas	Hemiepiphytic lianas	Hemiepiphytic lianas	Terrestrial/epiphytic shrubs
**Leaf texture**	Membranous	Chartaceous	Coriaceous	Membranous
**Leaf arrangement**	Clustered on adult branches	Alternate	Alternate	Clustered on adult branches
**Trichome type**	Simple, not glandular	Simple or branched, glandular or not glandular	Simple, glandular or not glandular	Simple, not glandular
**Inflorescence branching**	unbranched to forked	None; flowers solitary	Unbranched, forked or multi-branched	Unbranched
**Open flowers per inflorescence**	1–2	Solitary	(1)6–10 (>100)	4–6
**Peduncles**	Long (1–50 cm), hanging & slender	Absent	Short (0–3 cm), stout & woody	Intermediate (4–12 cm), hanging & slender
**Pedicels**	Unwinged or distally winged	Unwinged	Unwinged	Unwinged or distally winged
**Floral bract number and size**	0–2	Absent	Absent	2
	5–6 cm long × 1–2 cm wide			0.5–2 cm long × 0.1–0.3 cm wide
**Corolla symmetry**	Actinomorphic	Weakly zygomorphic	Weakly zygomorphic	Actinomorphic
**Corolla lobes**	Entire	Fimbriate	Fimbriate	Entire
**Corolla shape**	Infundibuliform or hypocrateriform to tubular-campanulate	Infundibuliform to cyathiform	Infundibuliform to cyathiform	Infundibuliform
**Corolla length**	8.5–15 cm	15–40 cm	2.5–21.5 cm	7–12 cm
**Fruit shape**	Conical	Conical	Globose, rarely conical	Conical or globose
**Fruit locule number**	2	4	2	2

## ﻿Materials and methods

All specimens of the tribe Solandreae from 25 Colombian and Ecuadorian herbaria were revised for the study to understand morphological variation across the group (acronyms follow Index Herbariorum http://sweetgum.nybg.org/science/ih/): ANDES, CAUP, COAH, COL, CUVC, FAUC, FMB, HECASA, HUA, HUAZ, HUQ, JAUM, JBB, JBGP, LLANOS, MEDEL, PSO, QCA, QCNE, TOLI, UDBC, UIS, UPTC, UTMC, and VALLE; as well as major herbaria that hold representatives from countries across the Andes (BM, E, F, K, MO, US). Herbarium material of the new species was collected in 2018 and deposited at Universidad del Tolima herbarium (TOLI) and Herbario del Jardín Botánico de Bogotá (JBB). Flowers and fruits were preserved in 70% alcohol to facilitate the preparation of taxonomic description and illustration.

Specimens with coordinates were mapped directly, and those lacking coordinates were located using Google Earth, GeoNames gazetteer (http://www.geonames.org), and GEOLocate Web service (https://www.geo-locate.org/default.html). Distribution maps were created using QGIS (QGIS Development Team 2021). Conservation assessments were made based on the IUCN Red List categories and criteria (IUCN 2012) and the most recent guidelines for using the IUCN Red List Categories and Criteria (IUCN 2022). For the conservation assessments, Extent of Occurrence (EOO) and Area of Occupancy (AOO) were calculated using GeoCat (www.geocat.kew.org; Bachman et al. 2011) with a 2 km^2^ cell size. Herbarium material, field observations, and photos were all used to construct the identification key.

## ﻿Taxonomic treatment

### ﻿ Doselia

Taxon classificationPlantaeSolanalesSolanaceae

A.Orejuela & Särkinen gen. nov.

B0EFD573-0D35-5F97-BA53-30525F85AC8F

urn:lsid:ipni.org:names:77302328-1

Fig. 1

#### Diagnosis.

Like *Solandra* Sw. and *Schultesianthus* Hunz., but differing from both in having membranous leaves (vs. chartaceous or coriaceous), lacking glandular trichomes, having pendulous inflorescences with long slender peduncles (vs. solitary flowers or short erect inflorescences with thick woody peduncles), distally winged pedicels, and actinomorphic corollas (vs. weakly zygomorphic); differing from *Solandra* in its smaller corollas (8.5–15 cm vs. 15–40 cm long), flowers borne in groups (vs. solitary), straight filaments and styles (vs. strongly curved and declinate), and 2-locular ovaries (vs. 4-locular); differing from *Schultesianthus* Hunz. in the chartaceous fruiting calyx with visible venation (vs. a thick, shiny and coriaceous fruiting calyx with no visible venation).

#### Type species.

*Doselialopezii* (Hunz.) A.Orejuela & Särkinen comb. nov. (basionym *Markealopezii* Hunz.)

#### Description.

Hemiepiphytic lianas adhering to the trees by adventitious roots. Stems terete when fresh, irregularly angulate when dry, pubescent with simple, uniseriate multicellular eglandular trichomes, older stems with pale brown and papery bark, often peeling, with broad circular foliar scars left by fallen leaves. Leaves alternate on young stems or tightly clustered appearing sub-opposite in adult branches, simple, broadly elliptic to obovate, membranous, concolorous, sparsely to densely pubescent both abaxially and adaxially with simple, uniseriate eglandular trichomes like those on stem; major veins 3–6 pairs; base attenuate to obtuse, sometimes asymmetric; margins entire to undulate; apex acute, acuminate, or mucronate; petioles well-developed, conspicuously articulate, green. Inflorescences axillary to sub-axillary monochasial cymes, simple to one-branched, occasionally bracteate, usually long-pedunculate and pendulous, 1–7-flowered, pubescent with trichomes as on the stems; peduncles (1.2–) 8.5–50 cm long; bracts absent or if present foliaceous and linear, 5–6 cm long, 1–2 cm wide; pedicels 0.5–3.0 cm long, conical, 5-ribbed, distally thickened and winged in some species, articulated at the base. Flowers 5-merous, actinomorphic, perfect, aestivation valvate in the calyx and cochlear in corolla. Calyx cupuliform, green to whitish-green with purple colouration sometimes on the veins or along the margins only; lobes flat to undulate, 2.4–5.2 cm long, 1–1.5 cm wide, long-triangular to lanceolate, apically long-acuminate to acute, pubescent with simple, uniseriate eglandular, transparent to brown trichomes. Corollas 8.5–15 cm long, the inner corolla diameter from 2.5–5.0 cm, infundibuliform, hypocrateriform to tubular-campanulate, orange, purple, white, yellowish-green with purple veins, sparsely pubescent abaxially with trichomes like those on the calyx; lobes 1.6–4.2 cm long, 1.6–4.3 cm wide, triangular to oblong, spreading to reflexed during anthesis, glabrous to sparsely pubescent, the margins entire to undulate to revolute, the apices acute to obtuse. Stamens 5, equal, included within corolla tube or fully exserted beyond the mouth; filaments 1.7–6.1 cm long, adnate to the base of the corolla, white to purple-tinged, glabrous to pubescent at the point of insertion; anthers 1.4–2.7 cm long, 1.3–1.8 mm wide, elongate, basifixed, not connivent. Ovary conical, 2.9–7.5 mm long, 2.9–7 mm in diameter, light yellow to brown, 2-carpellate, 2-locular, glabrous, with a well-developed 5-lobed light green to pale yellow nectariferous disc; style 5.9–8.8 cm long, straight, glabrous to sparsely pubescent with simple uniseriate trichomes 0.3–0.5 mm long; stigma 2-lobed, ca. 1 mm long and wide, usually clavate. Fruit a conical berry, 1.5–4.4 cm long, 1.5–4.4 cm in diameter, pale to dark green, chartaceous to coriaceous when dry, 2-locular, the exocarp 2–2.8 mm thick; fruiting calyx persistent, the lobes 3–5 cm long, 1.2–2.3 cm wide, appressed and enveloping the berry loosely, fully covering the fruit. Seeds numerous, 2.2–3.6 mm long, 1–1.7 mm wide, subreniform, the testa reticulate, the testa cells rectangular and straight in the outline, the embryo slightly curved, the cotyledons accumbent, slightly longer than the embryo rest, endosperm rather scanty. Chromosome number not known.

#### Etymology.

The generic name *Doselia* is derived from the Spanish word “dosel”, meaning canopy. It refers to the hemiepiphytic lianescent habit of all species of *Doselia*, with branches rising high up to the canopy to the top of tree crowns. The plants can be challenging to see because of their position on top of the tree canopy unless the plants have their showy pendulous flowers.

#### Distribution

**(Fig. 2).** Mid-elevation moist Andean forests from 500 to 2,300 m in Ecuador (Provinces of Morona Santiago, Napo, Pastaza) and Colombia (Departments of Antioquia, Boyacá, Caldas, Caquetá, Huila, Putumayo, Risaralda, Santander, Tolima, Valle del Cauca).

#### Discussion.

*Doselia* represents a morphologically distinct group of four hemiepiphytic lianas from mid-elevation moist Andean forests with very long branches extending to the forest canopy through adventitious roots. The combination of hemiepiphytic lianescent habit, membranous leaves arranged in tight clusters on adult branches, indumentum consisting of only simple eglandular trichomes, showy actinomorphic flowers arranged in elongated, pendulous, and few-flowered inflorescences, and conical fruits is unique within the tribe.

Within Solandreae, the lianescent hemiepiphytic habit is also known in *Solandra* and *Schultesianthus*, with the rest of the tribe mainly being epiphytic or rarely terrestrial shrubs (*Markeaantioquiensis* clade; Table 1). Leaves of all *Doselia* species are highly clustered on branch tips in whorls of 4–6 similar to species in the *Markeaantioquiensis* clade and some species of *Markea* (e.g., *M.plowmanii* Hunz.) and differ from all other genera and species of the tribe where leaves are more spread apart and clearly alternate (Table 1). Leaves in *Doselia* are membranous with simple eglandular trichomes on both surfaces, a character shared with some species of the *Markeaantioquiensis* clade (e.g., *M.pilosa* S.Knapp; Table 1). In many other genera of Solandreae, the leaves are chartaceous (e.g., *Hawkesiophyton* Hunz., *Juanulloa* Ruiz & Pav., *Merinthopodium* Donn. Sm., *Solandra* and *Trianaea* Planch. & Linden) or subcoriaceous to coriaceous (e.g., *Schultesianthus*) and often have simple glandular and/or dendritic trichomes in addition to the simple eglandular ones (Table 1).

Inflorescences in *Doselia* are long and pendulous (up to 50 cm long), with up to three flowers of which only one or rarely two develops at a time (Table 1). Such inflorescences are not typical in the tribe but are observed only in a few other species in Solandreae, including *Markeacoccinea* Rich., *Merinthopodiumneuranthum* (Hemsl.) Donn.Sm., *Merinthopodiumpendulum* (Cuatrec.) Hunz., and *Trianaeanobilis* Planch. & Linden. Pedicels in some *Doselia* species are distally winged because the sutures of the calyx are winged and continue onto the pedicel. Distally winged pedicels are also known in some species of the *Markeaantioquiensis* clade (e.g., *Markeaantioquiensis* S.Knapp and *Markeapilosa* S.Knapp; Table 1).

Corollas in *Doselia* are actinomorphic and showy, similar to species of the *Markeaantioquiensis* clade, but these two groups can be distinguished based on other characters such as growth form, peduncle length, number of open flowers per inflorescence, and floral bract and calyx size (Table 1). The two groups also differ in their calyx lobes, where lobes have acute to long-acuminate tips in *Doselia* but are rounded in the *Markeaantioquiensis* clade. Corollas in the two other morphologically closely related genera *Solandra* and *Schultesianthus* are slightly zygomorphic (Table 1).

Fruits in *Doselia* are conical, leathery, and fully covered by the calyx, like those of *Solandra*, but differ from the latter in being 2-carpellate and 2-locular, in contrast to the 2-carpellate and 4-locular fruits in *Solandra* (Table 1). Fruits in *Schultesianthus* appear similarly leathery but are globose in shape and covered only partially by an irregularly splitting calyx (Table 1). Chromosome number is not known for *Doselia*, but count numbers in other members of Solandreae, have shown a basic chromosome number ×=12 for *Dyssochroma* Miers (Piovano 1989; Acosta and Moscone 2000), *Solandra* (Campin 1924; Lepper 1982) and *Trianaea* (Chiarini et al. 2019). Similar chromosome counts might be expected for *Doselia*, but further research is necessary to confirm this assumption.

### ﻿Key to the species of *Doselia*

**Table:** 

1	Sparse pubescence throughout the plant, on the leaves confined to the veins and margins only, leaves glabrescent with age; style pubescent along its entire length	***D.galilensis***
–	Dense pubescence throughout the plant, on the leaves extending to the blade mesophyll, leaves persistently pubescent with age; style glabrous or with only a few trichomes at the very base	**2**
2	Anthers included within the corolla tube; corolla pale purple or purplish-cream; style with a few trichomes at the base	***D.epifita***
–	Anthers partially to entirely exserted beyond the corolla tube; corolla yellowish-green, brownish-green or orange; style completely glabrous	**3**
3	Corolla tubular-campanulate, the tube yellowish-green with a conspicuous reticulum of purple veins on both surfaces; anthers completely exserted	***D.huilensis***
–	Corolla infundibuliform, the tube orange with a conspicuous reticulum of purple veins at the base on the adaxial side only; anthers partially included	***D.lopezii***

### ﻿Species descriptions

### ﻿1. Doselia epifita

Taxon classificationPlantaeSolanalesSolanaceae

(S.Knapp) A.Orejuela & Särkinen comb. nov.

27D33022-8875-547F-83DC-034D337ED435

urn:lsid:ipni.org:names:77302329-1

Figs 1A, B , 3

Markea epifita S.Knapp, Novon 8(2): 155–157, f. 3a, b. 1998. Type. Ecuador. Napo: Canton Archidona, carretera Hollín-Loreto km 25, sector Challua Yacu, faldas al S de Volcán Sumaco, 1°45'S, 77°38'W, 1,200 m, 21 Apr 1989 (fl), *C. Cerón & F. Hurtado 6534* (holotype: QCNE; isotypes: MO! [MO-289398, acc. # 5343691], NY! [00214503]).

#### Type.

Based on *Markeaepifita* S.Knapp

#### Description.

Hemiepiphytic liana with adventitious roots. Stems sparsely pubescent with simple, uniseriate 2–4-celled hyaline trichomes ca. 0.5 mm long, giving the stems a tuberculate look. Leaves tightly clustered towards the branch tips, 11–25 cm long, 6–12 cm wide, obovate, sparsely pubescent with simple uniseriate 2–4-celled trichomes ca. 0.5 mm long, the trichomes denser and stiffer abaxially along the veins on both surfaces; major veins 4–5 pairs, not raised abaxially, drying dark brown; base attenuate, symmetric; margins entire; apex acute to acuminate; petiole 1–5 cm long, pubescent with stiff trichomes abaxially like those of the leaves. Inflorescence 18.5–45 cm long, axillary, unbranched, ebracteate, ca. 4–5-flowered, densely pubescent with simple, uniseriate trichomes like those of the stems; peduncle 8.5–35.2 cm long; pedicels 1.2–1.7 cm long, winged. Calyx 3.8–4 cm long, 1.4–1.5 cm wide, green tinged with purple, pubescent with simple uniseriate trichomes 0.5–1 mm long; tube 0.5–0.7 cm long; lobes undulate, 3.0–3.3 cm long, 0.8–1 cm wide, long-triangular, apically acuminate, densely pubescent abaxially with simple uniseriate trichomes 0.5–1 mm long, adaxially similar but also with tiny brownish papillate trichomes. Corolla 9–11 cm long, the inner corolla diameter 3–3.5 cm, infundibuliform, gradually widening from the base, glabrous; tube 7.5–8 cm, cream to green with lines or patches of purple; lobes 3.4–4.2 cm long, 2.5–3.3 cm wide, ovate, purple or violet, reflexed at anthesis, the margins slightly undulate, the apex obtuse to rounded, glabrous or with a few minute trichomes along the veins. Stamens 3.6–7.7 cm long, included within corolla tube; filaments 2–5 cm long, adnate to ca. 1 cm from the base of the corolla tube, white to purple-tinged, glabrous; anthers 1.6–2.7 cm long, 1.7–1.8 mm wide, cream. Ovary 3.3–3.4 mm long, 3.3–3.4 mm wide, colour unknown, glabrous; style 5.5–6 cm long, cream, glabrous except for a few simple uniseriate trichomes ca. 0.5 mm long at the very base; stigma clavate. Fruit not known. Chromosome number not known.

#### Distribution

(Fig. 2). On the eastern slopes of the Andes in central Ecuador (Provinces Morona-Santiago, Napo, and Pastaza) and Colombia (Departments Putumayo and Caquetá).

#### Ecology.

In premontane forest between 500–1,500 m elevation.

#### Preliminary conservation status

(IUCN 2022). Our data support the assessment of the species by Knapp et al. (2017) who considered *D.epifita* as vulnerable (VU) based on the criteria B1ab [iii]. *Doseliaepifita* is known from a few collections in the Cordillera de los Guacamayos, the protected areas of Sumaco-Napo-Galeras and Sangay, areas near the city of Puyo in Ecuador, the Natural Reserve “La isla escondida” in Putumayo, and the surroundings of the Alto Fragua indiwasi National Park in Caquetá, Colombia. The biggest threat to the species is deforestation (Knapp et al. 2017).

#### Discussion.

**Figure 3.**
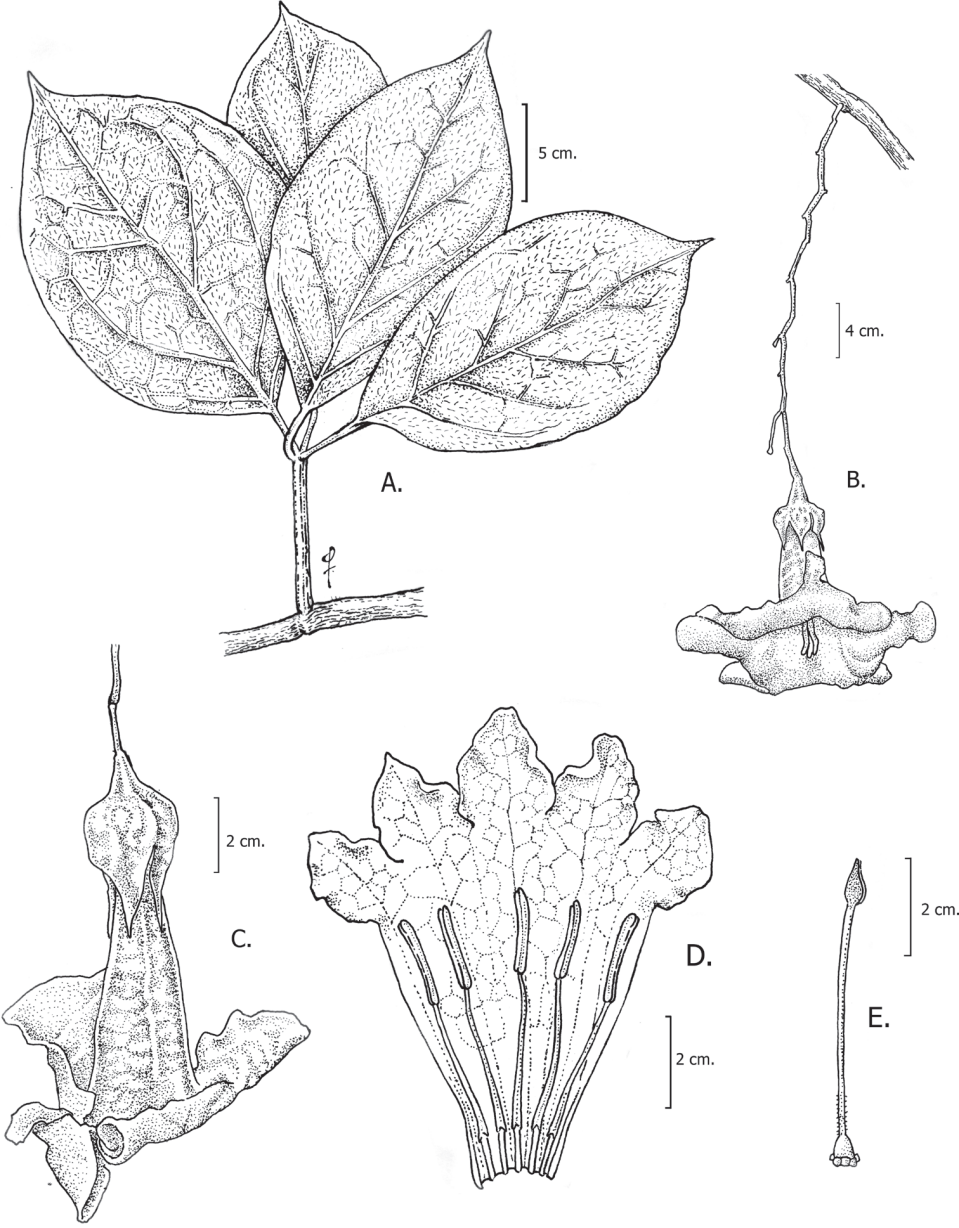
*Doseliaepifita***A** branch with clustered leaves **B** floriferous branch **C** flower at anthesis **D** dissected corolla showing inserted stamens **E** detail of gynoecium with a five-lobed nectariferous disc and a clavate stigma (**A–E***Ceron & Hurtado 6534*, *Palacios 6893*: Drawing by Omar Bernal).

*Doseliaepifita* is the only species of *Doselia* that reaches Ecuador and has the lowest elevational range within the genus. *Doseliaepifita* is morphologically most similar to *D.galilensis*, and a detailed comparison is presented under the latter. The inflorescence morphology of *D.epifita* was unknown until recently because no complete specimens with entire inflorescences were known when the species was first described (Knapp 1998). Recent collections have revealed that the inflorescences are axillary and long (18.5–45 cm long; Fig. 3B), as correctly predicted by Knapp (1998). The fruits of this species remain unknown.

#### Etymology.

The specific epithet refers to the apparent epiphytic habit of the species, though, like other species in the genus, *D.epifita* is a hemiepiphyte rather than an obligate epiphyte.

#### Specimens examined.

Colombia. **Caquetá**: Belén de Los Andaquíes, camino Andaquí, via que comunica Acevedo con Belén de los Andaquíes, vegetación a orilla de quebrada, 12 Mar 2016 (fl), *Cárdenas 45979* (COAH). **Ecuador. Morona-Santiago**: Parque Nacional Sangay, lagunas de Sardinayacu, 2°05'54"S, 78°09'19"W, 1,400–1,500 m, 18 Jan 2015 (fl), *Pérez et al. 7875* (QCA). **Napo**: Canton Archidona, Carretera Hollin–Loreto, km 25, Sector Challua Yacu, Faldas al sur del Volcán Sumaco, 1°27'00"S, 77°22'48"W, 1,200 m, 26 Aug 1980 (fl), *Cerón 6534* (MO, NY); Cantón Loreto, Parque Nacional Sumaco, Napo-Galeras, Matorral de Bambú, Bloque 19, línea sísmica 22, Compañía Triton, 0°47'00"S, 77°28'00"W, 500 m, 25 Mar 1996 (fl), *Freire & Cerda 271* (MO, QCNE); Cantón El Chaco, Proyecto Hidroeléctrico Coca, Punto ST3, margen derecha del Río Quijos, 10 km al S de Reventador, 0°11'00"S, 77°39'00"W, 1,500 m, 3 Oct 1990 (fl), *Palacios 5893* (MO). **Pastaza**: Cantón Mera, Carretera al Rio Ansu, 5 km al NE de Mera, 1°15'36"S, 78°03'36"W, 1,200 m, 16 Mar 1985 (fl), *Palacios et al. 123* (MO); Cantón Mera, Colonia Pindo, Mirador en la Reserva Pindo, 1°27'23"S, 78°04'47"W, 12 Nov 2011, *Orozco et al. 3876* (COL); Road Veracruz (Indillama) – Canelos, 1°35'00"S, 77°51'00"W, 25 Jun 1968 (fl), *Lugo 75* (MO); Cantón Pastaza, Shell, Río Pindo, 1°29'59"S, 78°03'44"W, 1,050 m, 18 Aug 1992 (fl), *Palacios 10380* (MO).

### ﻿2. Doselia galilensis

Taxon classificationPlantaeSolanalesSolanaceae

A.Orejuela & Villanueva sp. nov.

443B892E-448C-5416-BE29-C9E27E5600EE

urn:lsid:ipni.org:names:77302330-1

Figs 1C , 4 , 5

#### Diagnosis.

Differing from all other members of *Doselia* in its mature leaves with sparse pubescence of trichomes on the midvein and along margins only (vs. on entire lamina). Like *D.epifita* (S.Knapp) A.Orejuela & Särkinen but differing in the pale green to purplish-green calyces with flat lobes (vs. calyces green with purple patches with undulate lobes) and larger corolla 12–15 cm long (vs. 9–11 cm long).

#### Type.

Colombia. **Tolima**: Municipio Villarrica, Vereda Galilea, Bosque de Galilea, zona Campo Hermoso, junto a parcela permanente de Monitoreo de 1 ha, 03°46'21"N, 74°39'56"W, 1,543 m, 11 Jun 2018 (fl, fr), *L. Corrales*, *B. Villanueva*, *K. Sánchez & H. Díaz 917* (holotype: JBB! [JBB34413]; isotype: TOLI [TOLI26800]).

#### Description.

Hemiepiphytic liana with adventitious roots. Stems sparsely pubescent with simple, uniseriate 4–7-celled, hyaline trichomes 0.4–1.3 mm long, becoming glabrescent with age. Leaves tightly clustered towards the branch tips, 9.2–17.5 cm long, 6.4–8.4 cm wide, ovate to elliptic, sparsely pubescent with a few simple trichomes like those on the stems distributed along the margins and veins on both surfaces, especially on the young growth, glabrescent with age; major veins 3–4 pairs, slightly raised abaxially; base cuneate or obtuse, symmetric or rarely asymmetric; margins entire; apex acuminate to mucronate; petiole 0.8–1.8 cm long, sparsely pubescent with a few simple trichomes like those on the stems, glabrescent with age. Inflorescence axillary, simple, ebracteate, 11.5–17.2(–44) cm long, 1(–3)-flowered, sparsely pubescent with a few simple trichomes like those on the stems; peduncle 1.2–5.7(–32.5) cm long; pedicels 0.5–1.8 cm long, distally winged and thickened. Calyx 3.7–3.8 cm long, 1.7–1.8 cm wide, pale green with purple margins and reticulation along the veins, sparsely pubescent with simple, uniseriate trichomes like those on the stems; tube 0.5–0.7 cm long; lobes flat, 2.4–3.0 cm long, 1.0–1.2 cm wide, short-lanceolate, apically acute. Corolla 12–15 cm long, the inner corolla diameter 3.5–4.0 cm, infundibuliform; tube 8.3–9.5 cm long, with a narrow base 1.4–1.9 cm long, 0.8–0.9 cm wide and a wide distal portion 7.6–7.7 cm long, 3.6–3.8 cm wide, greenish-white with subtle purple veins, glabrous or sparsely pubescent with a few simple uniseriate trichomes like those of the rest of the plant on the tube externally; lobes 3.2–3.8 cm wide, 2.8–3.1 cm long, ovate, greenish-white with bright purple patches within, reflexed at anthesis, the margins revolute, the apex obtuse, glabrous. Stamens 4.1–4.2 cm long, included inside the corolla tube; filaments 3.1–3.4 cm long, adnate at ca. 1.4–1.8 cm from the base of the corolla, white, densely pubescent with simple, uniseriate 4–7(–12)-celled, hyaline trichomes at the insertion point; anthers 1.6–2.1 cm long, 1.4–1.5 mm wide. Ovary 3.7(–5.4) mm long, 6.2–6.3 mm wide, light brown, glabrous; style 5.9–6.5 cm long, cream, sparsely pubescent with simple short 2–4-celled uniseriate trichomes ca. 0.3 mm long; stigma clavate. Fruit ca. 4.4 cm long, ca. 2.9 cm wide, light green, the exocarp 2.1–2.4 mm thick, coriaceous and light yellow when dry; fruiting calyx persistent, accrescent and covering the fruit, enveloping the berry loosely, the lobes to 4–4.5 cm long, 1.3 cm wide. Seeds numerous, 3.3–3.6 mm long, 1.5–1.7 mm wide, ochre yellow when dry, the testa reticulate, the testal cells rectangular in outline, the embryo slightly curved, the cotyledons accumbent, slightly longer than embryo rest, endosperm rather scanty. Chromosome number not known.

#### Distribution

**(Fig. 2).***Doseliagalilensis* occurs in the western slopes of the eastern cordillera of the Colombian Andes and is only known from three localities in the municipality of Arcabuco (Department of Boyacá), the natural reserve “Reinita Cielo Azul” (Department of Santander) and the Parque Natural Regional Bosque de Galilea (Department of Tolima).

#### Ecology.

Grows in Andean tropical cloud forest from 1,500 to 2,300 m elevation.

#### Preliminary conservation status

(IUCN 2022). *Doseliagalilensis* is considered Data Deficient (DD) due to the small number of known populations. Based on our field observations, the biggest threat to the species is habitat loss due to agricultural expansion near the known localities. The situation has been alarming in the Galilea Forest during the last few years, with several direct threats to forest conservation such as agricultural expansion, unsustainable logging, and oil exploitation activities. Fortunately, the Galilea Forest has been recently declared as a protected area through the Corporación Autónoma Regional del Tolima (“CORTOLIMA” resolution 31 adopted on December 16, 2019). The Arcabuco oak forests in Boyacá do not, however, have any legal protection. It is unclear whether the new species remains in the area based on our unsuccessful attempt to collect *D.galilensis* in Arcabuco in 2019. The third population recently discovered in Santander is under the protection of the Proaves NGO in the natural reserve “Reinita Cielo Azul”.

#### Phenology.

*Doseliagalilensis* has been collected in flower in May, June and October and with fruits in June.

#### Etymology.

The epithet “*galilensis*” is in honour of the recently created “Parque Natural Regional Bosque de Galilea”, where the type specimen was collected. The Galilea Forest is located between 3°53'36"N, 74°31'51"W and 3°40'32"N, 74°44'20"W in the municipalities of Villarrica and Dolores. We hope that the description of this new Colombian endemic species highlights the importance of the Galilea Forest and stimulates more researchers to explore this beautiful reserve. The Galilea Forest covers more than 26,000 hectares and occupies an elevational range from 1,480 to 3,080 m. It represents a mid-elevation Andean montane forest sandwiched between the lowland tropical rain forest and treeline. Besides the typical Andean cloud forest, the Galilea Forest comprises cushion mire wetlands known as “turberas” and white-sand forests with species adapted to grow in these highly specialised soil conditions (e.g., *Utricularia* L., Lentibulariaceae). The Galilea Forest is considered a strategic ecosystem for water regulation in the watershed area of the Negro River and the Aco and Lusitania ravines that feed the Hidroprado Dam (Quimbayo-Cardona et al. 2019).

#### Discussion.

In the area of Arcabuco, Boyacá, *D.galilensis* is sympatric with *Merinthopodiumvogelii* (Cuatrec.) Castillo & R.E.Schult., a vegetatively similar species of Solandreae. *Merinthopodiumvogelii* differs in having green campanulate corollas with strongly reflexed lobes at anthesis and partially exserted anthers, while *D.galilensis* has included anthers and to greenish-white, infundibuliform corollas with slightly reflexed lobes that are purple-tinged at anthesis.

**Figure 4.**
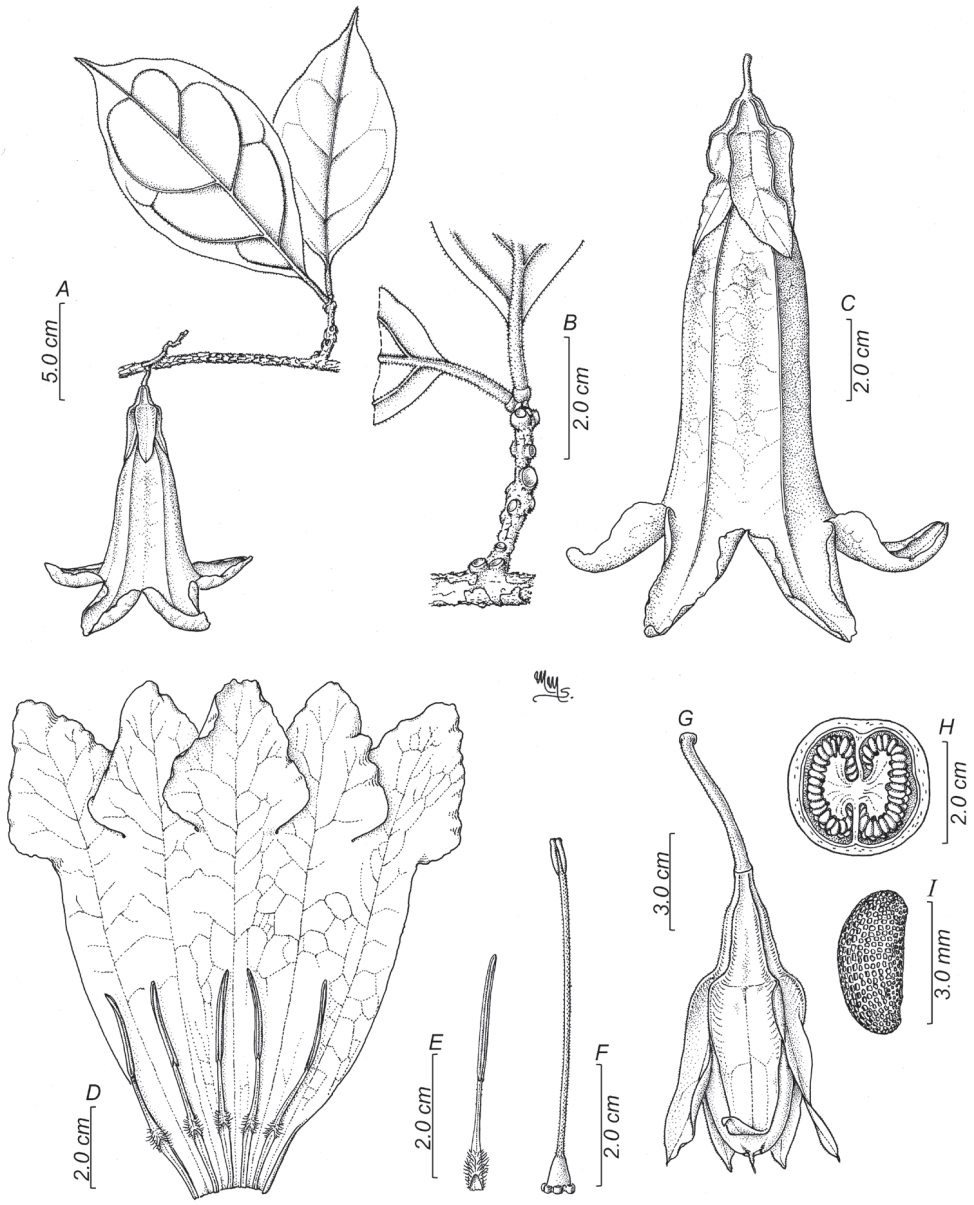
*Doseliagalilensis***A** floriferous branch **B** branch with clustered and circular foliar scars **C** flower at anthesis **D** dissected corolla showing inserted stamens **E** detail of a stamen **F** gynoecium with a five-lobed nectariferous disc **G** fruit with an accrescent calyx **H** dissected fruit showing placentation (cross-section) **I** seed (**A–I***Corrales et al. 917*: Drawing by Marcela Morales).

**Figure 5.**
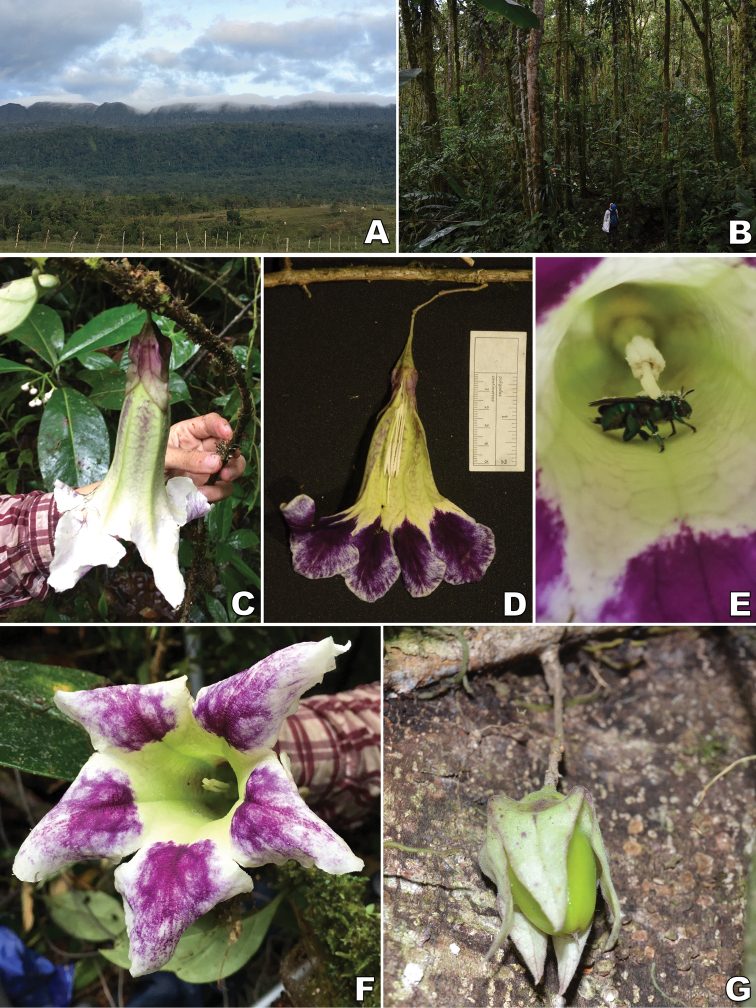
*Doseliagalilensis***A** habitat in type locality in Galilea forest, Villarrica, Tolima **B** mid-elevation moist forest habitat in Galilea forest **C** floriferous branch with a flower **D** floriferous branch with a dissected and opened corolla showing corolla colouration inside and the inserted stamens **E** female *Euglossa* bee visiting the flower **F** front view of the corolla showing the purple-tinged lobes and the inserted stamens **G** developing fruit covered by an accrescent calyx (**A–G***Corrales et al. 917*: Photographs by Boris Villanueva).

*Doseliagalilensis* can be easily differentiated from other species of *Doselia* in its glabrescent mature leaf blades where pubescence is sparse and restricted to midveins and margins (Fig. 1; Table 2). *Doseliagalilensis* is morphologically most similar to *D.epifita*; both species share several characters that are not present in other species of *Doselia*, such as infundibuliform corollas and included stamens with very short filaments (Fig. 1; Table 2). Unlike *D.epifita*, *D.galilensis* is sparsely pubescent, with only a few trichomes along the main veins of the leaves and very few trichomes in other parts of the plant. In contrast, *D.epifita* has a dense and persistent pubescence covering the entire plant with persistent trichomes on both sides of the leaves. The calyx lobes in *D.galilensis* are flat and lanceolate compared to the long-triangular undulate calyx lobes in *D.epifita*. *Doseliagalilensis* has slightly larger corollas with greenish-white tubes and purple-tinged lobes on the abaxial side (Fig. 5C–F) compared to *D.epifita* with white to purplish corolla tubes with purple lobes on both surfaces (Fig. 1B). Styles are consistently pubescent in *D.galilensis* along their entire length, while *D.epifita* has glabrous styles except for a few simple uniseriate trichomes at the very base.

**Table 2.** Morphological and geographical comparison of the four species assigned to *Doselia*.

	*D.epifita*	*D.galilensis*	*D.huilensis*	*D.lopezii*
**Distribution**	Napo, Pastaza (Ecuador), Putumayo, Caquetá (Colombia)	Boyacá, Santander, Tolima (Colombia)	Huila, Putumayo (Colombia)	Antioquia, Caldas, Risaralda, Valle del Cauca (Colombia)
**Elevation (m)**	500–1,500	1,500–2,300	2,200–2,300	1,700–2,100
**Leaf length × width (cm)**	11–25 × 6–12	9.2–17.5 × 6.4–8.4	9.0–16.7 × 4.6–11.7	14–22 × 4–9.8
**Leaf shape**	Obovate	Ovate to elliptic	Elliptic to broadly elliptic	Elliptic to broadly elliptic
**Mature leaf pubescence**	Sparsely pubescent on both surfaces with simple uniseriate trichomes	Sparsely pubescent on the main veins and margins with simple uniseriate trichomes, becoming glabrescent with age	Densely pubescent on both surfaces with simple uniseriate trichomes	Sparsely pubescent on both surfaces with simple uniseriate trichomes
**Peduncle length (cm)**	8.5–32.2	1.2–5.7(–32.5)	8.5–39	6–24(–39)
**Corolla shape**	Infundibuliform	Infundibuliform	Tubular-campanulate	Hypocrateriform
**Corolla length (cm)**	9–11	12–15	8.5–10	8–11
**Corolla lobe length × width (cm)**	3.4–4.2 × 2.5–3.3	3.2–3.8 × 2.8–3.1	2.3–3.3 × 1.6–1.7	3.2–3.9 × 3.7–4.1
**Anther position**	Included	Included	Exserted	Partially exserted
**Anther length (cm)**	1.6–2.7	1.6–2.1	1.4–1.9	1.9–2.2

#### Specimens examined.

Colombia. **Boyacá**: Municipio de Arcabuco, La Cumbre, 2300 m, 22 May 1980 (fl), *Pérez 01* (COL). **Santander**: Municipio de San Vicente de Chucurí, vereda Centro, sector Germania, parte alta, serranía de Los Yariguíes, camino de Lengerke entre Zapatoca y San Vicente de Chucurí, sector Reserva Proaves “Reinita Cielo Azul”, 6°50'46"N, 73°22'38W, 1672 m, 20 Oct 2021 (fl), *D. Díaz-Rueda et al. 2272* (JBB). **Tolima**: Municipio Dolores, Vereda El Carmen, Bosque de Galilea, zona Riachón, cerca de parcela permanente de monitoreo de 1 Ha No 5, 03°40'53.10"N, 74°41'6.56"W, 2122 m, 08 Aug 2019 (fl) *Rivera et al. 26* (JBB); Municipio Villarrica, La Colonia, vereda La Colonia, Bosque de Galilea 03°52'20.61"N, 74°33'12.12"W, 2000 m, 10 Jan 2020 (fl), *M. F. Valencia & M. Rincón 308* (TOLI).

### ﻿3. Doselia huilensis

Taxon classificationPlantaeSolanalesSolanaceae

(A.Orejuela & J.M.Vélez) A.Orejuela & Särkinen comb. nov.

3E93FAEE-4275-5355-AC19-A924DCB23BB3

urn:lsid:ipni.org:names:77302331-1

Figs 1D, E , 6

Markea huilensis A.Orejuela & J.M.Vélez, Phytotaxa 167(2): 156, Figs 6, 7. 2014. Type. Colombia. Huila: Municipio de La Plata, vereda La María, Finca Meremberg, sitio Agua Bonita, 02°12'13"N, 76°06'33"W, 2,287 m, 5 Aug 2010 (fl,fr), *A. Orejuela & J.M. Vélez-Puerta 112* (holotype: COL! [COL000420611]; isotypes: COL! [COL000420613]).

#### Type.

Based on *Markeahuilensis* A.Orejuela & J.M.Vélez

**Figure 6.**
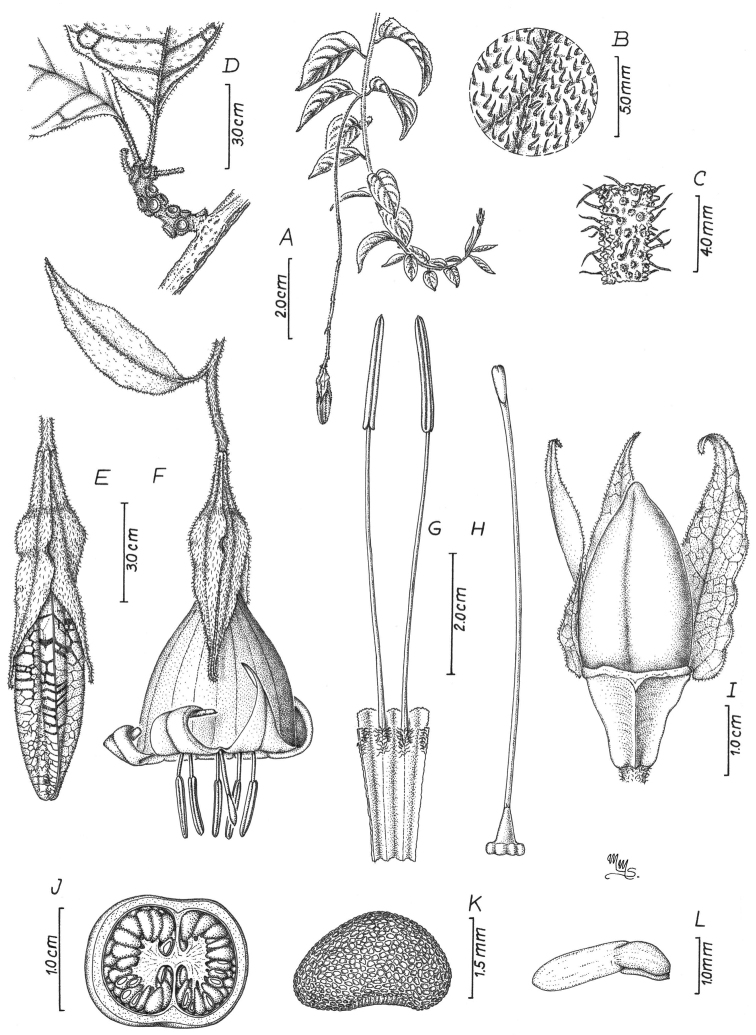
*Doseliahuilensis***A** floriferous branch **B** indument on the leaf blade **C** indument on young stems **D** leaf arrangement **E** flower bud **F** flower in anthesis **G** stamens **H** gynoecium **I** fruit with sepals removed **J** fruit in cross section **K** seed **L** embryo (**A–L***A. Orejuela & J.M. Vélez 112*: Drawing by Marcela Morales, first published in Orejuela et al. (2014), reproduced with permission).

#### Description.

Hemiepiphytic liana with adventitious roots. Stems densely pubescent with simple, uniseriate (2–) 4–7 (–11)-celled, hyaline to ochre-brown trichomes 0.2–1.8 mm long, with a deciduous apex and a persistent multicellular base giving the surface a tuberculate appearance, stems glabrescent with age. Leaves tightly clustered towards the branch tips, 9.0–16.7 cm long, 4.6–11.7 cm wide, elliptic to broadly elliptic, densely pubescent with simple 4–9-celled uniseriate hyaline to dark olive-brown trichomes 0.3–2 mm long on both surfaces; major veins 4–6 pairs, slightly raised abaxially; base cuneate or obtuse, asymmetric; margins entire to undulate; apex usually acuminate, mucronate; petiole 0.4–3.8 cm long, densely pubescent. Inflorescence sub-axillary, simple to branched, bracteate, 18–50 cm long, ca. 2–7-flowered, surface tuberculate and densely pubescent with trichomes as on the stems; peduncle 8.5–39 cm long; bracts foliaceous and linear, 5–6 cm long, 1–2 cm wide; pedicels 1.5–2 cm long, distally winged and thickened. Calyx ca. 3.3 cm long, 1.5 cm wide, dark green with purple margins and reticulate along the veins, pubescent with simple 4–7-celled uniseriate white hyaline to brown trichomes; tube 0.5–0.7 mm long; lobes undulate, 2.7–5.2 cm long, 1.3–1.5 cm wide, lanceolate, apically acuminate with an acumen 0.6–0.9 mm long, green with the main vein and the margins purple-brown, pubescent with simple uniseriate trichomes on the abaxial side. Corolla 8.5–10 cm long, the inner corolla diameter 4.5–5 cm, tubular-campanulate; tube 6.2–6.7 cm long, scarcely pubescent with trichomes similar to those of the calyx, yellowish green with strong purple-tinged reticulation along major and minor veins both abaxially and adaxially; tube differentiated into a narrow base ca. 0.2 cm long and 0.8–1 cm wide and a wide distal portion 4.2–4.6 cm long, ca. 5 cm wide; lobes 2.3–3.3 cm long, 1.6–1.7 cm wide, oblong, reflexed during anthesis, colour similar to that of the corolla tube, the margins revolute, the apex obtuse, glabrous. Stamens 6.1–6.9 cm long, fully exserted beyond corolla tube; filaments 4.7–5 cm long, adnate at ca. 2 cm from the base of the corolla, purplish, densely pubescent with simple uniseriate trichomes at the insertion point like those on calyx; anthers 1.4–1.9 cm long, 1.3–1.5 mm wide. Ovary ca. 7 mm long, ca. 3.5 mm wide, light yellow, glabrous; style 7.3–8 cm long, cream; stigma clavate. Fruit ca. 4.2 cm long, ca. 2.5 cm wide, dark green, exocarp 2–2.8 mm thick when fresh, coriaceous, black when dry; fruiting calyx persistent, accrescent and covering the fruit, appressed at maturity, the lobes 4–5 cm long, 2.2 cm wide. Seeds numerous, 2.6–3.0 mm long, 1.2–1.4 mm wide, ochre when fresh, dark brown when dry, the testa reticulate, the testal cells rectangular in outline. Chromosome number unknown.

**Figure 7.**
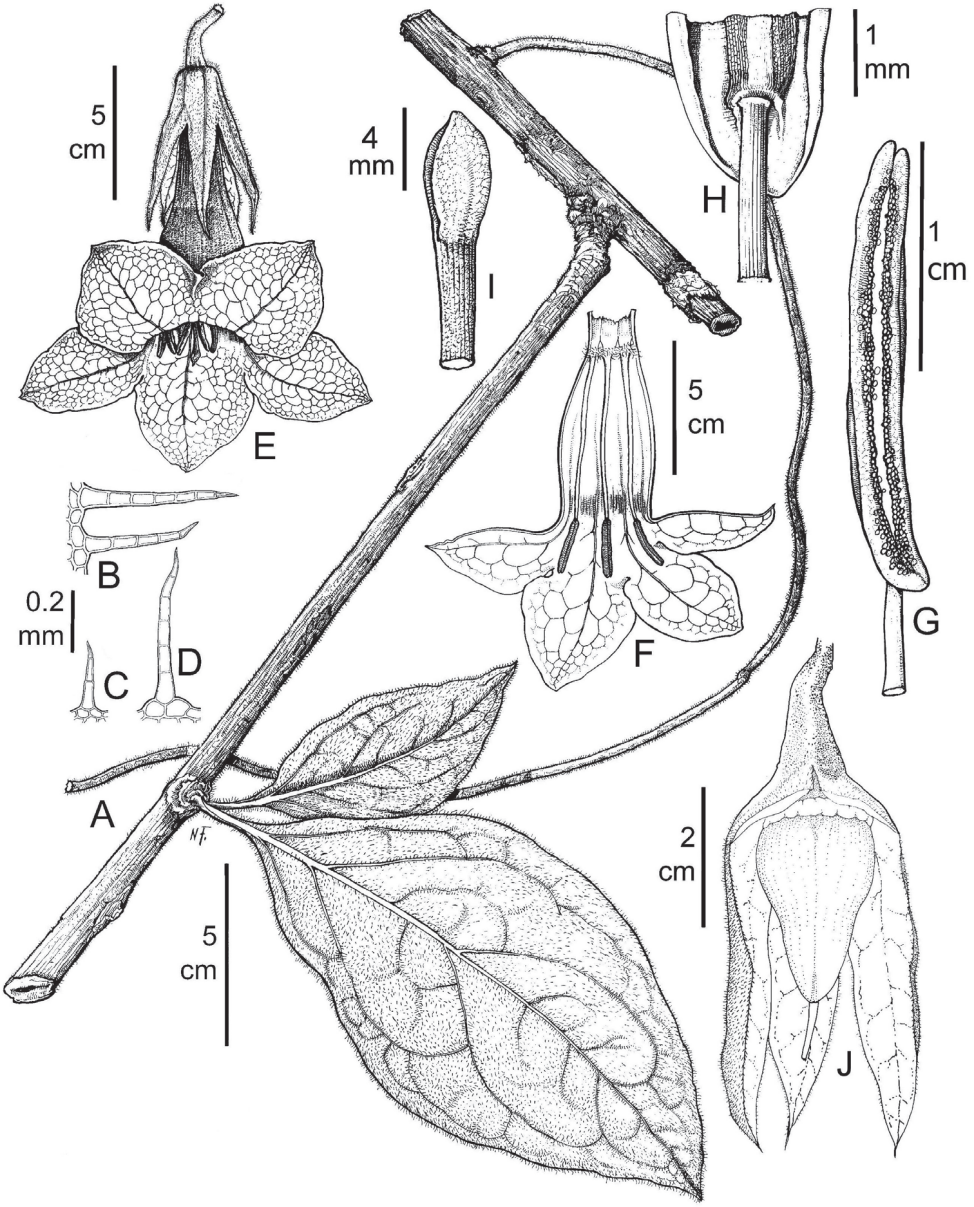
*Doselialopezii***A** branch with clustered leaves and an inflorescence axis **B** trichomes on leaves **C** trichomes on the calyx **D** trichomes at the filament insertion point **E** flower at anthesis **F** dissected corolla showing partially exserted stamens **G** stamen in lateral view **H** detail of a stamen showing the insertion of the filament **I** detail of the stigma **J** fruit with an accrescent calyx with two calyx lobes removed (**A–I***López Filgueiras 8208*: Drawing by Nidia Flury, first published in Hunziker (1985), reproduced with permission, the original drawing was edited by Omar Bernal and the fruit drawn by Humberto Mendoza).

#### Distribution

(Fig. 2). *Doseliahuilensis* is known only from the Departments of Huila and Putumayo in southwestern Colombia.

#### Ecology.

*Doseliahuilensis* is found in preserved or partially altered oak forests from 2,200 to 2,300 m elevation.

#### Preliminary conservation status

IUCN (2022). *Doseliahuilensis* is reaffirmed (following Orejuela et al. 2014) here as an endangered species (EN) according to criteria B1ab [i, iii] based on the small EOO (~750 km^2^), a small number of known populations, and the highly fragmented condition of the relictual forests where it occurs. The species is known from five collections from three localities. Two of these localities are in the Department of Huila 80 km apart, and one recent collection is known from the Valle del Sibundoy, Department of Putumayo, that extends the species distribution approximately 100 km to the south.

#### Discussion.

*Doseliahuilensis* differs from all other *Doselia* species in its tubular-campanulate corollas with fully exserted anthers (Table 2; Fig. 1D). The species is densely pubescent throughout, which is useful in distinguishing it from other *Doselia* species that are either glabrescent or less densely pubescent (Table 2).

#### Specimens examined.

Colombia. **Huila**: Municipio de La Plata,-vereda Agua Bonita, Finca Meremberg, 2,200–2,300 m, 21 Jul 1975 (fl), *Díaz-Piedrahita et al. 793* (COL); Carretera Popayán-La Plata, km 100, Reserva forestal de Fauna y Flora Meremberg, 2,300 m, 12 Dec 1982 (fl), *Murcia 09* (COL); Municipio San Agustín, vereda La Castellana, reserva privada Los Yalcones, interfluvio quebrada El Palmar-río Balseros, sitio El Palmar, 1°47´44"N, 76°21´5"W, 2,400–2,600 m, 15–20 Aug 2005, *Mendoza & Robles 16548* (FMB). **Putumayo**: Valle del Sibundoy, Reserva Natural Yumartán, cuenca alta del río Putumayo, por la garganta del Balsayaco, 3 Mar 2021 (fl), *Coral 34* (HEAA).

### ﻿4. Doselia lopezii

Taxon classificationPlantaeSolanalesSolanaceae

(Hunz.) A. Orejuela & Särkinen comb. nov.

60E31747-35C0-55E1-8770-778AB5AF5CCE

urn:lsid:ipni.org:names:77302332-1

Figs 1F–H , 7

Markea lopezii Hunz., Lorentzia 5: 9. 1985. Type. Colombia. Valle: Cuenca del Rio Cali, cercanías de Peñas Blancas, 10–11 Jan 1963 (fl), *M. López-Figueiras 8208* (holotype [two sheets]: US! [00385918, acc. # 2451166; 00385936, acc. # 24511165]).

#### Type.

Based on *Markealopezii* Hunz.

#### Description.

Hemipiphytic liana with adventitious roots. Stems sparsely pubescent with simple, uniseriate 4–8-celled, hyaline ochre trichomes 0.5–1.2 mm long, with deciduous apex and a persistent multicellular base giving the surface a tuberculate appearance, stems glabrescent with age. Leaves tightly clustered towards the branch tips, (7–)10–22 cm long, 4–9.8 cm wide, elliptic to broadly elliptic, sparsely pubescent with simple uniseriate 4–7-celled hyaline trichomes 0.8–1.2 mm long on both sides; major veins 4–6 pairs, slightly raised abaxially; base cuneate, slightly asymmetric; margins entire; apex acute to acuminate; petiole (7–)10–19(–25) mm long, densely pubescent with simple trichomes as on the leaves. Inflorescence axillary, simple, ebracteate, 7–35(–50) cm long, ~2–5-flowered, densely pubescent with simple, uniseriate trichomes like those on stems; peduncle 6–24(–39) cm long; pedicels 0.8–1.7(–3.0) cm long, distally winged and thickened. Calyx 3.8–5.6 cm long, 1.4–1.9 cm wide, green, sometimes tinged with purple, densely pubescent with simple, uniseriate trichomes like those on leaves; tube 8–9 mm long; lobes flat, 3.5–4.4 cm long, 1.2–1.4 cm wide, long-triangular, apically long-acuminate, green or green with purple margins, sparsely pubescent with simple uniseriate trichomes on the abaxial side. Corolla 8–11 cm long, the inner corolla diameter 2.5–2.8 cm, hypocrateriform; tube 7–10 cm long, orange and tinged with purple in the throat internally, glabrous; lobes 3.2–3.9 cm long, 3.7–4.1 cm wide, triangular, orange, spreading to slightly reflexed at anthesis, the margins flat and entire, the apex acute, glabrous or with a few minute trichomes along the veins. Stamens 7–8.6 cm long, partially exserted beyond the mouth of corolla tube; filaments 5.3–6.1 cm long, adnate at 1.0–1.3 cm from the base of the corolla tube, purple-tinged, densely pubescent with simple 6–10-celled uniseriate trichomes at the insertion point; anthers 1.9–2.2 cm long, 1.4–1.8 mm wide. Ovary ca. 7.5 mm long, ca. 2.9 mm wide, yellow, glabrous; style 7.9–8.8 cm long, glabrous; stigma clavate. Fruit 2.7–3.2 cm long, 1.5–3.3 cm wide, light green; fruiting calyx persistent, accrescent and fully covering the fruit, the lobes 3–3.3 cm long, 1.2–1.4 cm wide. Seeds numerous, 2.2–2.7 mm long, 1–1.3 mm wide, ochre when fresh, brown when dry, the testa reticulate, the testal cells rectangular in outline. Chromosome number not known.

#### Distribution

(Fig. 2). *Doselialopezii* is endemic to the pre-montane forests of the Colombian Andes in the departments of Antioquia, Caldas, Valle del Cauca and Risaralda.

#### Ecology.

Mid-elevation moist forests from 1,700 to 2,100 m elevation.

#### Preliminary conservation status

(IUCN 2022). *Doselialopezii* is classified as vulnerable (VU) according to the B1a criterion with an EOO of ca. 6,000 km^2^. The area where it is distributed is severely fragmented and the species is known from fewer than ten localities..

#### Discussion.

*Doselialopezii* is the type species of the genus and the easiest species to recognise on account of its showy flowers with large orange corollas (Table 2; Fig. 1F, G). *Doselialopezii* has anomalous and apparently unique pollen in the genus with prominent spiny supratectal processes (Persson et al. 1994). Preliminary observations in *D.huilensis* (Orejuela et al. 2014) and specimens of *D.epifita* examined by Hunziker (1997, as *M.lopezii*) indicate that the pollen of these two species lack these spiny supratectal processes.

#### Specimens examined.

Colombia. **Antioquia**: Medellín– Puerto Triunfo, Cocorna, ca. 5 km E of Cocorna Peaje, Quebrada El Biadal, 6°N, 75°10´W, 1,830 m, 23 Nov 1983 (fl), *Juncosa 1400* (JAUM); Pulperies 6000, Jul 1880 (fl), *Kalbreyer 1638* (K, MO). **Caldas**: Municipio Samaná, corregimiento Florencia, Vereda San Vicente, sector río Claro - Sierra Morena, Parque Nacional Natural Selva de Florencia, 5°31'21"N, 75°3'40"W, 1,840 m, 2 Oct 2012 (fl), *Betancur et al. 16698* (COL). **Risaralda**: Municipio de Pueblo Rico, en cercanías de los límites con el PNN Tatamá, Reserva Santuario-Tatamá, 5°11'03.1"N, 76°01'16.5"W, 1821 m, 18 Mar 2022 (fl), *Orejuela et al. 3849* (JBB). **Valle del Cauca**: Finca Torremolinos, km 22, carretera entre Cali y Buenaventura, cordillera occidental, vertiente occidental, 1,800 m, 13 Oct 1982 (fl), *Albert de Escobar et al. 2678* (HUA); km 18, vía Cali–Buenaventura, chemin lateral, 1,920 m, 8 Mar 1997 (fl), *Billiet & Jadin 6904* (MO); Estación Microondas Tokio, 8 km W of Queremal (along old road to Buenaventura), 2°27'00"N, 76°45'00"W, 26 Sep 1980 (fl), *Croat 50164* (MO); Hoya del río Dígua, Quebrada del San Juan, subiendo a Paragüita desde Queremal, 3°32'09"N, 76°42'42"W, 1,570–1,740 m, 17 Mar 1947 (fl), *Cuatrecasas 23830* (F); Hacienda Tokio, wet montane forest behind microwave tower ca. 10 km S of Queremal, 3°30'00"N, 76°42'00"W, 2,000 m, 26 Feb 1983 (fl), *Gentry et al. 40820* (MO); La Cumbre, vereda La Sofía, corregimiento de Bitaco, Cerro de Yumbillo, cordillera occidental, vertiente occidental, 1,850 m, 6 Dec 1988 (fl), *Klimkiewicz & Cabrera 275* (CUVC); Cuenca del río Cali, cercanías de Peñas Blancas, 3°26'10"N, 76°38'28"W, 10 Jan 1963 (fl), *López-Filgueiras 8208* (US); La Cumbre, corregimiento de Bitaco, vereda Chicoral, 3°33'56"N, 76°35'3"W, 2,020 m, 23 Jul 2003 (fl), *Mendoza et al. 15275* (FMB); km 18, vía Cali–Buenaventura, vereda Dapa, Finca Zíngara, 1,800 m, 6 Nov 2009 (fl), *Orejuela 59* (COL); vía Cali–Dagua, km 23, Reserva Privada El Refugio, 4 Nov 2011 (fl), *Orejuela & Calderón-Sáenz 170* (COL); Vía Cali-Dagua, km 23, Reserva Privada El Refugio, 3°32'02"N, 76°36'56"W, 1,870 m, 13 Feb 2014 (fl), *Orejuela et al. 727* (COL, JBB).

## Supplementary Material

XML Treatment for Doselia

XML Treatment for Doselia epifita

XML Treatment for Doselia galilensis

XML Treatment for Doselia huilensis

XML Treatment for Doselia lopezii
